# National genomic survey of drug-resistance and multi-jurisdictional clusters of *Mycobacterium tuberculosis* in Australia 2015–2023

**DOI:** 10.1016/j.lanwpc.2026.101928

**Published:** 2026-07-23

**Authors:** Jessica R. Webb, Eby Sim, Kristy Horan, Caitlin Selway, Patiyan Andersson, Susan Moss, Stanley Pang, Arnold Bainomugisa, Alireza Zahedi, Justin T. Denholm, Elena Martinez, Sushil Pandey, Maria Globan, Taryn Crighton, Ella M. Meumann, Dimitrios Menouhos, Robert W. Baird, Norelle L. Sherry, Karina Kennedy, Louise Cooley, Lex Leong, Clare Slogget, Torsten Seemann, Christopher Coulter, Amy V. Jennison, Vitali Sintchenko, Benjamin P. Howden, Australian Pathogen Genomics program

**Affiliations:** a Microbiological Diagnostic Unit Public Health Laboratory, The University of Melbourne at the Peter Doherty Institute of Infection and Immunity, Melbourne, Victoria, Australia; b Department of Microbiology and Immunology at The Peter Doherty Institute for Infection and Immunity, The University of Melbourne, Melbourne, Victoria, Australia; c Department of Microbiology and Immunology, Centre for Pathogen Genomics, University of Melbourne, Victoria, Australia; d Microbiology and Infectious Diseases, SA Pathology, Adelaide, South Australia, Australia; e Public and Environmental Health Reference Laboratories, Pathology Queensland, Queensland Department of Health, Brisbane, Queensland, Australia; f Sydney Infectious Diseases Institute, School of Medical Sciences, Faculty of Medicine and Health, The University of Sydney, Sydney, New South Wales, Australia; g New South Wales Mycobacterium Reference Laboratory, Institute of Clinical Pathology and Medical Research, NSW Health Pathology, Sydney, New South Wales, Australia; h Territory Pathology, Royal Darwin Hospital, Darwin, Australia; i Department of Clinical Microbiology and Infectious Diseases, Canberra Health Services, Australian National University Medical School, Canberra, Australian Capital Territory, Australia; j Department of Microbiology and Infectious Diseases, Royal Hobart Hospital, Hobart, Tasmania, Australia; k Queensland Mycobacterium Reference Laboratory, Pathology Queensland, Queensland, Australia; l Pathogen Genomics and Surveillance, PathWest Laboratory Medicine, Nedlands, Western Australia, Australia; m Victorian Tuberculosis Program, Melbourne Health, at the Peter Doherty Institute for Infection and Immunity, Melbourne, Victoria, Australia; n Department of Infectious Diseases, The University of Melbourne, at the Peter Doherty Institute for Infection and Immunity, Melbourne, Victoria, Australia; o Victorian Mycobacterium Reference Laboratory (MRL), Victorian Infectious Diseases Reference Laboratory (VIDRL), at the Peter Doherty Institute for Infection and Immunity, Melbourne, Victoria, Australia; p Antimicrobial Resistance and Infectious Diseases Research Laboratory, Murdoch University, Perth, Western Australia, Australia; q School of Biological Sciences, Adelaide University, Adelaide, South Australia, Australia; r Global and Tropical Health Division, Menzies School of Health Research, Charles Darwin University, Darwin, Northern Territory, Australia; s Division of Medicine, Department of Infectious Diseases, Royal Darwin Hospital, Darwin, Northern Territory, Australia; t Department of Infectious Diseases & Immunology, Austin Health, Heidelberg, Victoria, Australia

**Keywords:** Tuberculosis, Genomics, Public health

## Abstract

**Background:**

Tuberculosis is a global public health priority. In Australia, genomic analysis of *Mycobacterium tuberculosis* (*Mtb*) occurs at state or territory level, thus multijurisdictional genomics of *Mtb* remains poorly understood. We present the first national assessment of *Mtb* using nine years of genomic data from all Australian jurisdictions, as a part of the Australian Pathogen Genomics program.

**Methods:**

Our national *Mtb* sequence dataset spanned 2015–2023 and represented culture-confirmed cases. AusTrakka was optimised to allow secure, national contribution of *Mtb* genomes and metadata. Drug resistance was determined using *tbtamr*, and genomic clusters were identified via hierarchical single linkage clustering (5 SNPs) from pairwise SNP distances. Criteria were established to identify clusters of national public health significance.

**Findings:**

We analysed 6616 Mtb sequences, representing 59% of notified cases nationwide. Lineages 1–4 were detected, with lineage 2 the most prevalent and drug resistant. At the 5-SNP threshold, 373 genomic (17% of sequences) clusters were identified, with 48 (13%) clusters identified as nationally significant. Critically, 31 genomic clusters spanned multiple jurisdictions and persisted for >5 years. Genomic analysis inferred low rates of *Mtb* drug resistance in Australia.

**Interpretation:**

This first-of-a-kind study on genomic diversity of *Mtb* in Australia highlighted the benefits of national aggregation and analysis of *Mtb* sequencing data. It identified multi-jurisdictional clusters which, on average, persisted longer than clusters within jurisdictions. We also established the framework and infrastructure for implementing national coordination, detection, and surveillance of priority clusters to enhance tuberculosis control in Australia.

**Funding:**

Australian National Health and Medical Research Council, Medical Research Futures Fund (FSPGN00049).

**Research in context**

**Evidence before this study**

The utility of whole genome sequencing of *Mycobacterium tuberculosis* has been pivotal in surveillance and transmission tracking in high-income, low incidence settings. Australia is a federation of two territories and six states, each with their own regional TB control programs. PubMed searches using the terms “*M. tuberculosis*”, “genomics”, “Australia” and “sequencing”, returned genomic epidemiology snapshot reports from four of eight jurisdictions, each focused on their own localised settings. A comprehensive genomic survey at the national level, has yet to be undertaken.

**Added value of this study**

This study collected and curated a set of genomic sequences of *M. tuberculosis* from all eight jurisdictions and provided a comprehensive analysis of the genomic landscape in Australia spanning 2015 to 2023. To our knowledge, this is the largest dataset of *M. tuberculosis* genomes from a single country. While low rates of genotypic drug resistance were identified, our analysis uncovered the possibility of protracted genomic clusters that spanned multiple jurisdictions. These clusters have gone unnoticed with genomic surveillance efforts primarily concentrated at the individual jurisdictional level. Additionally, this study also provided a framework for national genomic surveillance of *M. tuberculosis* which will be utilised in future genomic surveillance of TB in Australia.

**Implications of all the available evidence**

Our findings highlighted the need for continued and concerted national monitoring of *M. tuberculosis* to ensure that Australia meets its aim of local TB elimination. The data presented, along with our proposed framework for genomic surveillance provides the foundation to inform public health policy for the use of genomics to control TB in Australia. Additionally, our proposed framework could also be considered by other federated countries without a routine centralised genomic surveillance workflow for pathogens of public health concern.

## Introduction

*Mycobacterium tuberculosis* (*Mtb*) is the causative agent of tuberculosis (TB), with an estimated 10.8 million new cases of TB in 2023 with low- and middle-income communities experiencing 80% of the total number of TB cases reported. The World Health Organization “TB elimination action framework for low incidence countries” defines pre-elimination as <10 TB cases/million population with the aim of reaching this by 2035,^1^ to which Australia has committed. Australia is a low TB incidence country (on average 5 cases of disease per 100,000) with ∼90% of TB cases occurring in people born overseas.^2^ Whilst pre-elimination has been achieved in non-indigenous Australian born populations, targets have not been achieved in Aboriginal and Torres Strait Islanders,^2^ who experience four to five times the TB disease burden compared to non-Aboriginal and Torres Strait Islander Australians.^3^ A multi-pronged approach is needed to achieve pre-elimination targets in First Nations Australians and culturally and linguistically diverse populations. Sustainable genomic surveillance of *Mtb* has potential to assist in identifying local transmission networks, allowing for early public health interventions.^4^

In Australia, TB is a notifiable disease and 88% of cases are confirmed by the isolation of *Mtb* in culture or by detection using nucleic acid testing such as GeneXpert (Cepheid).^3^^,^^5^ Whole genome sequencing (WGS) can provide improved turnaround-time and better accuracy in identifying DR-TB in comparison to phenotypic drug susceptibility testing (pDST), which is still the gold-standard. Furthermore WGS, offers high resolution tracking of TB transmissions and outbreak clusters,^6^^,^^7^ highlighting added value of WGS for TB case management.

Australia consists of two territories and six states (henceforth collectively referred to as jurisdictions) with varying TB incidence, each with individual public health units and TB control programs supported by diagnostic and public health laboratories (PHLs). There are five state Mycobacterium Reference Laboratories (in New South Wales (NSW), Queensland (QLD), South Australia (SA), Victoria (Vic) and Western Australia (WA)) that provide pDST services as well as WGS of *Mtb* isolates for all jurisdictions in Australia. However, genomic and epidemiological analysis of *Mtb* occurs at a jurisdictional level, with different levels of capacity for integration into public health. Studies within Australian jurisdictions have identified limited transmission in the south-eastern jurisdictions (VIC and NSW),^4^^,^^6^, ^7^, ^8^ whilst northern jurisdictions (QLD)^9^(Bainomugisa et al., 2024) and the Northern Territory (NT) exhibit more persistent transmission networks spanning decades.^10^ However, despite apparent differences across the country, there have been no national *Mtb* genomic studies performed Australia-wide. In 2021 the Australian Pathogen Genomics Program (AusPathoGen) was established to implement national genomic surveillance for pathogens of public health concern in Australia. *Mtb* was identified as a priority pathogen within Australia and one of the first key pathogens of the AusPathoGen research program to assess the utility of genomics for enhanced national surveillance.

In close collaboration with jurisdictional PHLs and Health Departments and the National Tuberculosis Advisory Committee (NTAC) and clinicians, we undertook Australia’s first national genomic assessment to determine the feasibility and potential utility of national *Mtb* surveillance. To directly address this, we readied AusTrakka^11^ (the Australian government endorsed platform for real-time genomic data sharing for outbreak investigations) for national TB surveillance. Using an extensive *Mtb* genomic dataset from all jurisdictions across nine years, we undertook a unified analysis to investigate genomic drug-resistance determinants and establish whether multi-jurisdictional genomic clusters were present.

## Methods

### Ethical approval

Ethics approval was obtained from the Royal Melbourne Hospital Human Research Ethics Committee (study number HREC/83761/MH-2022) under the Australian National Mutual Acceptance Scheme, with subsequent recognition by authorised Human Research Ethics Committees from all participating jurisdictions.

### Setting and study design

The AusPathoGen national *Mtb* working group (WG) (65 members from 24 organisations who held 22 national meetings from 2022 to 2024) was formed to develop and manage the AusPathoGen *Mtb* projects.^12^ The metadata variables and sequence datasets were defined by the WG members and included one dataset with two sampling strategies spanning 9-years from 2015 to 2023. These datasets encompassed an eight-year (2015–2022) retrospective dataset to capture temporal trends and a second prospective dataset consisting of a 12-month snapshot (2023) dataset, whereby all Australian jurisdictions attempted to sequence all culture-positive cases of *Mtb*. 7093 sequences were contributed from across Australia, with collection dates of original clinical specimens between 2015 and 2023 (inclusive). 6616 sequences from across the country passed sequence QC (Supplementary methods) and were available for lineage assignment, cluster identification, and assessment of genomic AMR. Relevant sample (including date of collection) and demographic (limited to jurisdiction of case residence, age and gender) metadata were defined by the national *Mtb* WG members and contributed by data custodians.^12^ Details of the isolates included in this study are available in Supplementary Table S1. In consultation with the NTAC, genomic clusters of national significance were defined as cases which clustered at the 5 SNP threshold and fulfilled any of the following criteria: (i) ≥ 5 cases from ≥ 2 jurisdictions (not MDR); (ii) ≥10 cases from a single jurisdiction (not MDR); (iii) ≥ 2 case within a cluster with genomic markers of MDR-TB.

### Establishment of AusPathoGen repository in AusTrakka for *Mtb*

A specific project was established in the AusTrakka platform for *Mtb* so that genome sequences and metadata could be shared by jurisdictional PHLs and Departments of Health and aggregated in one location. Access to *Mtb* sequences and metadata is restricted to those individuals who are formally part of AusPathoGen and includes sequence and metadata custodians, bioinformaticians, epidemiologists and researchers who develop and manage the projects.

### National server and bioinformatic analysis

A dedicated AusPathoGen sever was set up so that bioinformaticians could login from across Australia to undertake analysis collaboratively across the country. Sequence quality control and data analysis was designed and performed by a multidisciplinary team of bioinformaticians, genomic epidemiologists and researchers that is made up of six individuals from the larger *Mtb* WG spanning all jurisdictions^12^ (Supplementary Methods).

### Statistical analysis

To determine if national *Mtb* genomic surveillance could provide additional insights into *Mtb* populations recovered from patients across multiple jurisdictions, genomic cluster size, duration and intra-cluster distances were compared between clusters where only one jurisdiction was involved and where multiple jurisdictions were involved. Due to the number and unequal numbers of clusters and skewed distributions, significance was assessed using a Mann–Whitney test,^13^ as implemented in Python statistical package scipy v 1.15.2.^14^

### Figures and data visualisation

All figures and plots were created in with Python (v 3.11.11), using following packages: pandas v2.2.3^15^ and numpy v2.2.2^16^ for data analysis and altair v5.5.0^17^ for generation of data visualisation.

### Role of funding source

The funders of the study had no role in study design, data collection, data analysis, data interpretation, or writing of the report.

## Results

### Contribution of sequences from across Australia

**Fig. 1:**
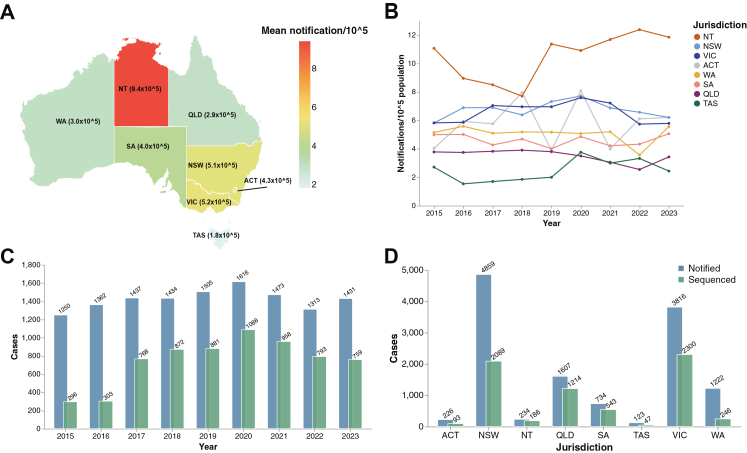
**Notifications and sequences of *Mtb* in Australia during the study period (2015–2023). A)** The average number of notifications per 10ˆ5 of population from 2015 to 2023 for each Australian jurisdiction **B)** The number of notifications per 10ˆ5 population per year for each Australian jurisdiction from 2015 to 2023. **C)** Number of notified cases sequenced in Australia from 2015 to 2023. **D).** Total number of sequences per notified cases per jurisdiction from 2015 to 2023.

Australia is considered a low *Mtb* incidence country, with fewer than 2000 cases notified nationally per year. However, the less populated NT has on average the highest notification rate compared to other Australian jurisdictions, with 8–12 notifications per 100,000 population per year (Fig. 1A and B). More populated states in Eastern Australia such as VIC and NSW have higher numbers of notifications, but proportionally lower notifications per population size.

Sequence data was contributed by all jurisdictions, although only QLD, NT and SA contributed sequences for 2015 and 2016 (Supplementary Fig. S1). From 2017 to 2019 all jurisdictions, except WA, contributed sequences for at least 10% notified cases (Supplementary Fig. S1). Whilst from 2020 to 2023 all jurisdictions were able to contribute sequences for 62% of notified cases (Supplementary Fig. S1). Overall, across the entire study period we collected sequences for 59% of notified cases nationally (Fig. 1C and D).

### Temporal dynamics of global lineages in Australia

**Fig. 2:**
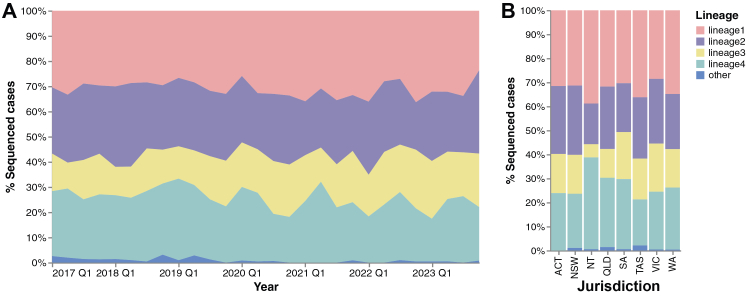
***Mtb* lineages represented in the Australian sequence dataset.** Lineages were identified and expressed as the percentage of each lineage per sequences available **A)** per year and **B)** per jurisdiction. The 4 major global lineages are reported, <10 sequences nationally were identified for lineage 5,6 and 9 and a small percentage of mixed lineages (represented by blue, other).

All the major global *Mtb* lineages (Lineage 1–4) were represented in the Australian dataset (Fig. 2). The distribution of lineages remained consistent over the study period, with the most common lineage being lineage 1 comprising 30% of sequences (2033/6616) and lineage 4 the least common comprising 16% sequences (Fig. 2). The distribution of lineages in the NT were distinct from the other jurisdictions, with proportionally more lineage 1 and lineage 4 sequences observed (Fig. 2B and Supplementary Fig. S2). Other jurisdictions exhibit similar distributions of lineages to each other.

### National *Mtb* drug resistance survey

Antimicrobial resistance mechanisms in *Mtb* have been extensively studied, with the WHO catalogue providing a basis for high confidence prediction of drug resistance profiles from genomic sequence data.^18^ This current study does not aim to recapitulate any of these high-quality reports, but rather characterise the genomic resistance trends in Australian *Mtb*. Resistance to *Mtb* is categorised as resistance profiles, such as susceptible, mono-resistant, MDR etc (Supplementary Table S1). Detection of genomic anti-mycobacterial mechanisms and classification of drug resistance was undertaken with tbtamr,^19^^,^^20^ which has been extensively validated and accredited to ISO standards using the WHO v2 catalogue.^21^

**Fig. 3:**
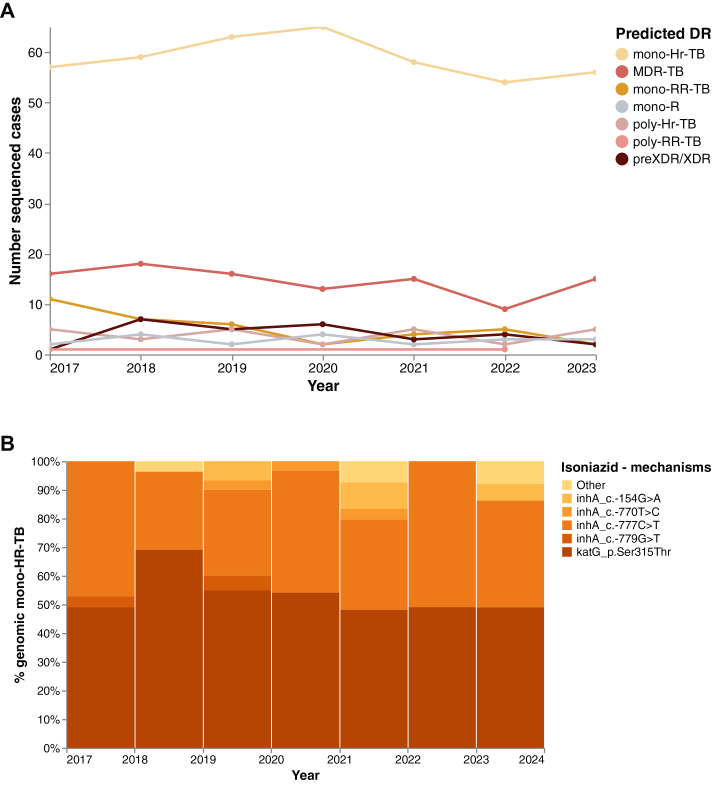
**Genomic AMR profiles represented in the Australian sequence dataset. A)** Genomic drug resistance detected by year (2017–2023). **B)** Temporal breakdown of isoniazid resistance mechanisms by year (2017–2023).

The most common resistance mechanisms observed in the Australian sequence collection confer isoniazid resistance (632/6616 sequences - 9.6%), these mechanisms can be seen mostly in mono-Hr-TB (71.5% 452/632 observations of isoniazid resistance), but also with lower frequency in MDR-TB (19% 120/632 observations of isoniazid resistance) and preXDR/XDR-TB (4.7% 30/632 observations of isoniazid resistance). This trend was mostly due to the presence of the common and well characterised mutation, *katG*_p.Ser315Thr and mutations in the promoter of *inhA*, predominantly at position −777C>T (Fig. 3B). Resistance to other first-line antimycobacterial drugs occurs at much lower frequency in the Australian dataset, with resistance mechanisms to rifampicin observed in only 2.9% (192/6616 sequences) of sequences. Unlike isoniazid, the majority rifampicin resistance was seen in MDR-TB (62.5% 120/192 observations of rifampicin resistance), with mono-RR-TB making up only 20.9% of the observations of rifampicin resistance (40/192 observations) (Table 1). Genomic markers of ethambutol and pyrazinamide resistance were found in 1.9% and 1.4% respectively. Resistance mechanisms to moxifloxacin was also very low, occurring in only 1.5% of the sequences (Table 1). Most of these resistance mechanisms were identified in the context of no resistance to first-line drugs (65.3% 66/101 observations of moxifloxacin resistance). However, 29.7% of these moxifloxacin resistance observations occurred in the presence of rifampicin and/or isoniazid resistance (30/101 observations) and were reported as preXDR/XDR. Resistance mechanism for newer drugs for treatment of Mtb, such as bedaquiline, are not as well characterised as the other drugs, however recent improvements to the WHO catalogue mean that some mechanisms can be identified from genomic sequences.^21^ We identified 14 sequences with mechanisms that may cause resistance to bedaquline, 11 of these were in the presence of no first-line resistance, however 2 were seen in sequences otherwise categorised as MDR-TB (Table 1).

**Table 1:** *Mtb* genomic DST by drug and lineage (2015–2023).

gDST	Lineage 1 30.7% (2033)	Lineage 2 26.4% (1749)	Lineage 3 25.3% (1671)	Lineage 4 16.7% (1107)	Other lineages 0.9% (56)	Total (n = 6616)
Rifampicin						
Total	1.4% (29)	6.3% (111)	1.3% (22)	2.5% (28)	3.6% (2)	2.9% (192)
Resistance profile	mono-RR (9) MDR (20)	mono-RR (18) poly-RR (1) MDR (68) preXDR/XDR (24)	mono-RR (4) MDR (14) preXDR/XDR (4)	mono-RR (8) poly-RR (1) MDR (17) preXDR/XDR (2)	mono-RR (1) MDR (1)	mono-RR (40) poly-RR (2) MDR (120) preXDR/XDR (30)
Isoniazid						
Total	10.8% (219)	14.6% (256)	4.0% (67)	7.5% (84)	10.7% (6)	9.6% (632)
Resistance profile	mono-Hr (190) poly-Hr (9) MDR (20)	mono-Hr (149) poly-Hr (15) MDR (68) preXDR/XDR (24)	mono-Hr (49) MDR (14) preXDR/XDR (4)	mono-Hr (60) poly-Hr (5) MDR (17) preXDR/XDR (2)	mono-Hr (4) poly-Hr (1) MDR (1)	mono-Hr (452) poly-Hr (30) MDR (120) preXDR/XDR (30)
Ethambutol						
Total	0.8% (17)	4.6% (71)	1.0% (16)	1.8% (20)	0% (0)	1.9% (124)
Resistance profile	mono-R (2) poly-Hr (4) MDR (11)	mono-R (1) poly-Hr (8) poly-RR (1) MDR (43) preXDR/XDR (18)	mono-R (3) MDR (10) preXDR/XDR (3)	mono-R (1) poly-Hr (5) poly-RR (1) MDR (12) preXDR/XDR (1)		mono-R (7) poly-Hr (17) poly-RR (2) MDR (76) preXDR/XDR (22)
Pyrazinamide						
Total	1.1% (22)	2.9% (50)	0.2% (4)	1.3% (14)	1.8% (1)	1.4% (91)
Resistance profile	mono-R (9) Poly-Hr (5) MDR (8)	(mono-R n = 3, Poly-Hr-TB n = 7, MDR-TB n = 27, preXDR/XDR n = 13, Total n = 50)	mono-R (2) preXDR/XDR (2)	mono-R (5) Poly-Hr (1) MDR (7) preXDR/XDR (1)	poly-Hr (1)	mono-R (19) Poly-Hr (14) MDR (42) preXDR/XDR (16)
Ethionamide						
Total	7.7% (157)	6.4% (112)	1.1% (18)	3.8% (42)	1.8% (1)	5.0% (330)
Resistance profile	FL susceptible (12) mono-Hr (129) Poly-Hr (3) MDR (13)	FL susceptible (11) mono-Hr (61) Poly-Hr (2) MDR (27) preXDR/XDR (11)	FL susceptible (2) mono-Hr (13) MDR (1) preXDR/XDR (2)	FL susceptible (1) mono-Hr (31) Poly-Hr (2) Poly-RR (1) MDR (6) preXDR/XDR (1)	mono-Hr (1)	FL susceptible (26) mono-Hr (235) Poly-Hr (7) Poly-RR (1) MDR (47) preXDR/XDR (14)
Moxifloxacin						
Total	0.2% (4)	2.5% (44)	2.2% (37)	1.4% (16)	0% (0)	1.5% (101)
Resistance profile	FL susceptible (4)	FL susceptible (17) mono-Hr (1) mono-RR (2) preXDR/XDR (24)	FL susceptible (31) mono-Hr (2) preXDR/XDR (4)	FL susceptible (14) preXDR/XDR (2)		FL susceptible (66) mono-Hr (3) mono-RR (2) preXDR/XDR (30)
Amikacin						
Total	0.5% (11)	0.8% (14)	1.0% (17)	1.0% (10)	0% (0)	0.8% (52)
Resistance profile	FL susceptible (10) mono-R (1)	FL susceptible (8) mono-RR (1) mono-Hr (1) MDR (3) preXDR/XDR (1)	FL susceptible (14) mono-Hr (2) preXDR/XDR (1)	FL susceptible (8) mono-RR (1) preXDR/XDR (1)		FL susceptible (40) mono-R (1) mono-Hr (3) mono-RR (2) MDR (3) preXDR/XDR (3)
Bedaquiline						
Total	0.05% (1)	0.3% (5)	0.4% (7)	0.1% (1)	0% (0)	0.2% (14)
Resistance profile	FL susceptible (1)	FL susceptible (2) mono-Hr (1) MDR (2)	FL susceptible (7)	FL susceptible (1)		FL susceptible (11) mono-Hr (1) MDR (2)

Sequences were assessed for the presence of genomic DST mechanisms for rifampicin, isoniazid, ethambutol, pyrazinamide, ethionamide, amikacin and bedaquline.

Header row provides breakdown of lineages (%, total). Results are presented as a percentage of observations for each lineage. Brackets contain the number of sequences which fall into each drug resistance profile (as determined by WHO guidelines) and the total number of sequences observed with any resistance mechanism to the drug assessed. The final column indicates the total percentage of sequences in the Australian dataset with resistance to each drug. The resistance profile reflects the drug resistance profile for Mtb as per the WHO guidelines [REF] (*FL susceptible* = no resistance mechanisms detected to first-line anti-mycobacterial drugs, rifampicin, isoniazid, ethambutol or pyrazinamide; *mono-RR* = mono-resistance to rifampicin; *mono-Hr* mono-resistance to isoniazid; *mono-R* = mono-resistance to either pyrazinamide OR ethambutol but NOT rifampicin or isoniazid; *ploy-RR* = resistance to rifampicin and pyrazinamide and/or ethambutol but NOT isoniazid; *poly-Hr* = resistance to isoniazid and pyrazinamide and/or ethambutol but NOT rifampicin; *poly-R* = resistance to ethambutol and pyrazinamide and/or isoniazid OR rifampicin; *MDR* = resistance to rifampicin and isoniazid with or without resistance to ethambutol or pyrazinamide; *preXDR/XDR* = resistance to rifampicin and moxifloxacin with or without resistance to other anti-mycobacterial drugs.

### Persistent multi-jurisdictional genomic clusters identified in Australia

We undertook analysis to investigate the potential for ongoing local transmission of *Mtb* across the jurisdictional borders of Australia. Lineages where <5 sequences were observed or sequences where mixed lineages were detected were excluded from the core-genome alignment, leaving 6591 sequences across lineages 1, 2, 3, and 4 for subsequent cluster analysis. Consistent with previous work, phylogenetic trees exhibit long branch lengths - with few clades containing closely related sequences (Supplementary Fig. S3).

Previous studies have shown that using a 5 SNP threshold allows for identification of clusters which are more likely to represent current transmission networks.^6^^,^^21^ Using this threshold we identified 337 genomic clusters, encompassing 17.3% (1143/6591) of sequences. Most clusters identified were made up of genome pairs (68.5% (231/337)) and off these pairs, 168 pairs fell within a single jurisdiction, with the remaining 63 pairs identified in different jurisdictions. Lineage 2 and 4 had the highest percentage of sequences participating genomic clusters, despite lineage 1 being the most abundant lineage in the Australian dataset (Supplementary Fig. S6).

**Fig. 4:**
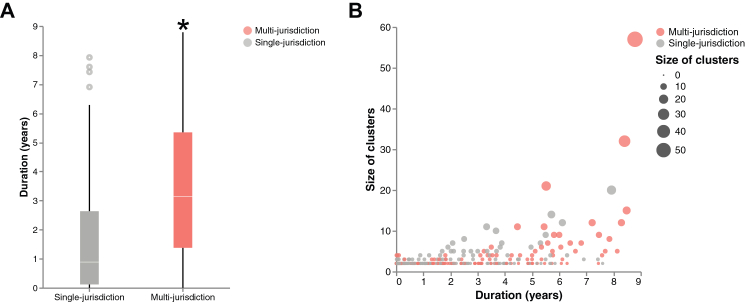
**Characteristics of the genomically identified Australian multi-jurisdictional clusters. A)** The duration of clusters which spanned 2 or more jurisdictions (light orange bar) was significantly higher than the duration of clusters which were restricted to single jurisdictions (Light grey bar) (∗ Mann–Whitney z = 18021.5, p < 0.001). **B)** The size of genomic clusters (0–50 sequences, size of circle) and duration of clusters in years was compared and coloured by jurisdiction if present in one jurisdiction or defined as multi-jurisdictional (MJ) if present in more than one jurisdiction.

**Fig. 5:**
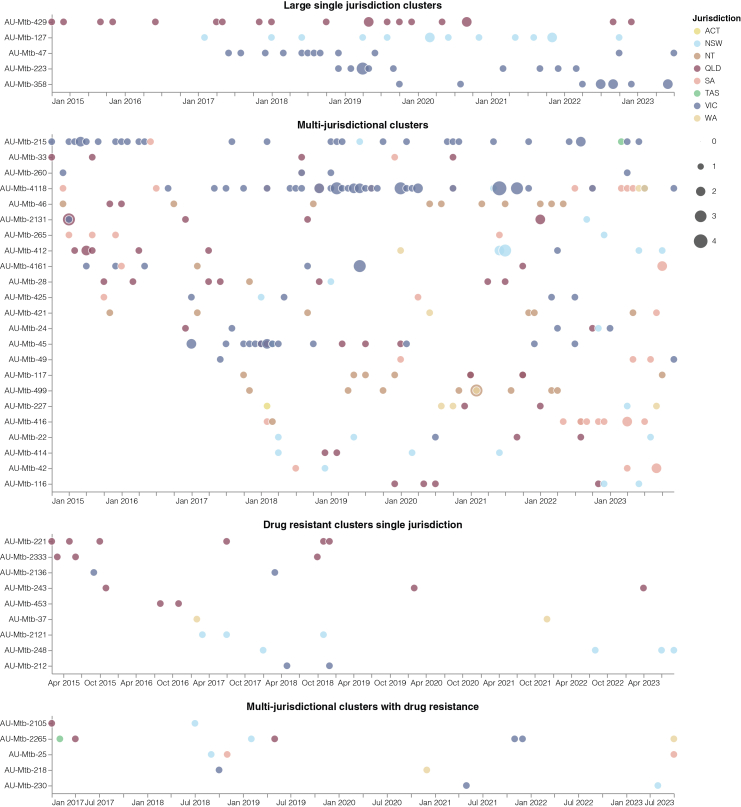
**Timeline plot of the Australian genomic clusters identified as nationally significant.** Genomic clusters of national significance were identified according to the 4 criteria and cases within each cluster was plotted by the date of collection of the cases and coloured by the jurisdiction. The y-axis represents the cluster ID assigned for the purposes of this study.

We identified 378 sequences across 42 clusters which were categorised as clusters of national significance (see Methods), corresponding to 5.8% of the available sequences. All jurisdictions had at least one case sequence that fell into a category of national significance (Supplementary Fig. S5). Of these 42 clusters, 14 (41 cases) exhibited evidence of drug-resistance, with a median of two cases per cluster. This represented 5.9% (41/699 sequences that with non-susceptible drug resistance profiles) of all drug resistant sequences in the Australian dataset. Five of these clusters were multi-jurisdictional with the largest comprised of 7 cases. Of the 14 drug-resistant clusters, 12 were from lineage 2 and 1 each belonged to lineage 3 and lineage 4. We also identified 23 multi-jurisdictional clusters with five or more cases, with clusters of lineage 4 being over-represented (52% 12/23) compared to the percentage of lineage 4 sequences in the Australian dataset (16% all sequences were lineage 4). We also identified five clusters which had 10 or more cases, but were confined to a single jurisdiction, the largest of which contained 20 sequences from QLD (Fig. 4). At least three large (>10 cases), multi-jurisdictional clusters spanned the entire study period (2015–2023). Cases belonging to these three multi-jurisdictional clusters ranged from 12 to 57 cases (AU-Mtb-215 n = 29, AU-Mtb-4118 n = 57 and AU-Mtb-46 n = 12). Additionally, clusters AU-Mtb-46 and AU-Mtb-412 appeared to shift from QLD to other jurisdictions over time (Fig. 5).

Multi-jurisdictional clusters (clusters containing ≥ 2 sequences) persisted over longer time periods with a median of 3.1 years, compared to the clusters that occurred within a single jurisdiction which had a median duration of 0.7 years (p < 0.001). Importantly, multi-jurisdictional clusters also tended to be larger in size, ranging in size from 2 to 57 cases compared to 2–20 cases for single jurisdictional clusters (Fig. 4). The contribution of lineages to single and multi-jurisdictional clusters also differed (Supplementary Fig. S7). Lineage 4 clusters comprised 25% of single jurisdictional clusters and contribute 25% of clustered sequences, but increases to 33.9% of multi-jurisdictional clusters, comprising 48.4% of the total number of sequences in multi-jurisdictional clusters (Supplementary Fig. S7).

Median intra-cluster SNP-distances were also larger in multi-jurisdictional clusters than those from single jurisdictions (median 5 IQR 1.5–10 versus median 0 IQR 0–0). However, these SNP-distances did not correlate with the size or duration of the cluster (Supplementary Fig. S7), with some of the largest and most persistent clusters having median SNP-distance of ≤2.

## Discussion

The AusPathoGen *Mtb* programme has established a large dataset of WGS data from clinical *Mtb* cases collected from across Australia’s eight jurisdictions over a 9-year period. Our dataset represents 59% of cases diagnosed between 2015 and 2023 and for the first time, has allowed for a national analysis of genomic relationships and AMR across Australia. Critically, we identified prolonged previously undetected clusters that occurred across jurisdictional boundaries – which we then used to inform development and implementation of *Mtb* genomic surveillance. The Australian *Mtb* collection, has representation from all four major global *Mtb* lineages with AMR profiles that remained constant across the study period. We observed very low numbers of genomically defined resistance, consistent with previous studies.^22^^,^^23^

Detection of AMR mechanisms in the Australian *Mtb* cohort remained low and unchanged across the study period. Isoniazid mono-resistance was the most common resistance pattern observed and was largely due to the well characterised mutations in *katG* (p.Ser315Thr) and promoter mutations in *inhA*. The prevalence of the genomic observations presented here for isoniazid resistance are consistent with the number of phenotypic observations from across Australia in the same time frame^22^ and therefore would not require re-assessment of treatment guidelines in Australia. The Australian *Mtb* population exhibited very low levels of MDR and preXDR/XDR cases, with resistance mechanisms for bedaquiline (13 observations) and linezolid (1 observation) occurring very rarely. Furthermore, the occurrence of drug-resistance in *Mtb* in Australia appears to be largely sporadic, with only 5.9% of sequences with a non-susceptible drug resistance profile being part of a genomic cluster. Despite this, ongoing genomic analysis will facilitate monitoring for emergence of resistance mechanisms that are linked to XDR-TB as the global knowledge of genomic mechanisms for these critical drugs expands.

Routine *Mtb* genomic surveillance programs have been established in the south-eastern states of Australia since 2017 and 2018 (NSW, VIC and SA). Other jurisdictions, such as QLD, have only recently begun to undertake genomic surveillance of *Mtb*, and retrospective sequencing studies in the NT that highlight the existence of persistent clusters within underserved communities^9^^,^^24^ have been undertaken. The current study indicates that many *Mtb* cases in Australia appear to be sporadic (i.e., not clustered) and likely due to independent importation from travel or migration from high-burden countries as opposed to local transmission. However, we also highlight that of the clusters identified, those within single jurisdictions are significantly shorter in duration and tend to be smaller in size than those which span multiple jurisdictions. In addition, there are differences between the single-jurisdictional and multi-jurisdictional clusters, in terms of lineage distribution that cannot be explained by simple persistence and therefore increased possibility of subsequent expansion into other jurisdictions. The specific drivers of this transmission will require detailed case level follow up and additional data collection, but the multi-jurisdictional nature of these clusters has not been apparent prior to this work. This may indicate that whilst jurisdictional TB control programs are effective in containment within their communities, ongoing multi-jurisdictional transmission pathways have not been investigated. This highlights how clusters that span boundaries would be more efficiently detected through a coordinated national surveillance program. Differing sequencing strategies were in place across Australia from 2015 to 2022, with some jurisdictions, such as SA, VIC and NSW establishing routine genomics sequencing of culture positive *Mtb*. Whilst other jurisdictions, such as WA sequenced more sporadically during this time. Smaller jurisdictions, such as ACT, TAS and NT were provided with sequencing capacity by other larger jurisdictions, although this was not uniform. This means, that it is possible that genomic clusters of *Mtb*, are not fully represented by this dataset. In practice, it is likely that not all *Mtb* will be sequenced, as not all cases are culturable, and not all culturable samples generate sequence data appropriate for inclusion in a comparative analysis. However, the current study demonstrates that it is possible to detect genomic clusters in such a dataset to provide actionable insights into potential multi-jurisdictional transmissions.

There are few documented instances of national genomics surveillance programs for *Mtb* globally.^25^, ^26^, ^27^, ^28^, ^29^ These reports primarily address the laboratory implementation aspects of *Mtb* WGS and/or are set up in settings within single jurisdictions/countries. Where single jurisdictions are involved, such as in Singapore, where genomic data is generated at a single site and reported centrally, sharing of genomic data and accompanying metadata is straightforward.^27^ However, sharing of data across jurisdictions in Australia is not routine and can be challenging, due to the federated nature of the public health system.^30^ Furthermore, each jurisdiction has its own Mycobacterial reference laboratory, most have their own sequencing capacity and report to the jurisdictional TB programs within their state. Different governance, permission structures and infrastructure are some of the challenges that can hamper the establishment of national surveillance for any pathogen in Australia. For *Mtb*, there are additional considerations, including the stigma associated with *Mtb* in some communities. This has implications for individuals as well as communities that are disproportionately impacted by *Mtb*, such as members of immigrant and Indigenous communities. Guidelines exist to respect, and to mitigate the risk to, Indigenous populations.^31^^,^^32^ However, these predominantly focus on research and sequencing of human genomic material in mechanistic research. Less has been done in regard to pathogen genomics and infectious diseases for clinical and public health. This presents different challenges due to the reasons for sampling and requirements for notification.^32^ Further research capturing enhanced risk factor data or rollout of national surveillance for *Mtb* in Australia will require consultation with community representatives to understand concerns and to co-develop strategies to ensure appropriate use of data and maintenance of appropriate data sovereignty.

The AusTrakka platform facilitates adherence to ethical and legal obligations of the jurisdictions. All sequence and metadata uploaded remain under the custodianship of the jurisdiction of the case and are managed by the appropriate organisations within that jurisdiction.^30^ All data is stored in secure cloud storage, with backups at multiple sites. Thus ensuring that only the appropriate personnel gain access to the data and that it is used in an appropriate way. The use of the AusTrakka platform further allows for central harmonised quality control and analysis and is undertaken on a dedicated public health server maintained by the national AusTrakka team, with limited access on a closed network. For the purposes of this initial research work, which focusses on the genomic relationships of *Mtb*, the use of specific epidemiological data identifying the possible underserved communities was not included.

**Fig. 6:**
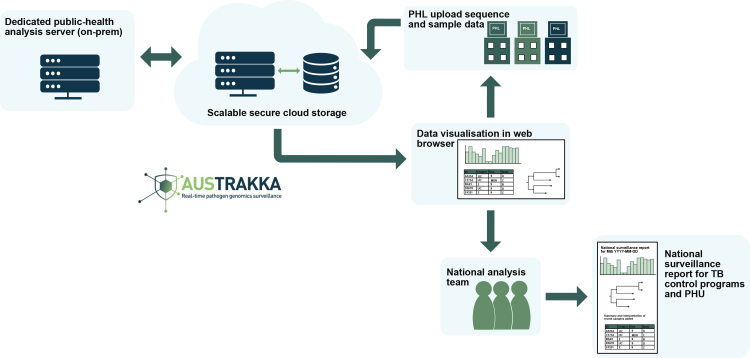
**Model for national genomic surveillance of *Mtb* in Australia**.

As a part of this project, public health stakeholders flagged that a consistent cluster nomenclature was essential to TB genomic surveillance implementation and communication. Therefore, following consultation with the AusTrakka National Analysis Team and public health agency stakeholders we developed a cluster nomenclature system which allows for a degree of consistency, whilst still being flexible to allow for the shifts in cluster membership that can arise when using hierarchical clustering to define genomic clusters (Supplementary Fig. S5). These genomic clusters will be assigned IDs in the following format: AU-Mtb-ClusterID. Based on the work outlined here by the AusPathoGen program, a national AusTrakka genomic surveillance program for *Mtb* has been proposed and is awaiting national endorsement. This national surveillance program does not aim to replace jurisdictional surveillance programs but to provide consistent and interoperable results at the national level to highlight possible multi-jurisdictional *Mtb* transmission for further investigation in real-time. Jurisdictions can prospectively upload WGS data monthly to the AusTrakka platform, where the data is analysed centrally. Results would be available in AusTrakka portal for public health laboratories to view in real time and reports written by domain experts within the AusTrakka National Analysis Team and communicated to the public health units and TB control programs (Fig. 6).

Although Australia is a high-income country with low TB incidence, it is important to recognise that *Mtb* disproportionately impacts underserved populations.^2^ Genomic surveillance is one tool that can potentially contribute to improved TB control in these populations, with the aim of reaching pre-elimination. The current study highlights the utility of centralised, harmonised multi-jurisdictional genomic analyses to identify potential transmission across jurisdictional boundaries, which could lead to improved public health responses and reduce the disparity between these populations. In addition, given the rise of MDR TB globally and in our region, prospective monitoring for transmission of drug resistance in Australia could prevent expansion of these strains. Further work is required to understand the epidemiology of these transmission networks to possibly identify case characteristics that can perhaps be used to better understand *Mtb* transmission and prioritise cases for sequencing. Further characterisation of the genomic epidemiology of the clusters identified here will be used to develop policy and improve jurisdictional and national analysis strategies. In addition, consultation with impacted communities will also further enhance the national surveillance of *Mtb* in Australia.

In conclusion, this first national genomic survey of *Mtb* in Australia highlighted the benefits of the national aggregation and analysis of sequencing data and has developed strategies for national co-ordination, implementation and ongoing monitoring of TB in Australia. We have identified several previously undetected multi-jurisdictional clusters which, on average, persisted longer than clusters within single jurisdictions. National coordination of detection and monitoring of priority and drug-resistance associated clusters will likely enhance tuberculosis control in Australia and minimise the risk of transmission events in a low-incidence country.

## Contributors

BH, VS, JRW, ES and KH designed the study. BH is the programme lead of AusPathoGen. Public Health Laboratories and Mycobacterium Reference Laboratories provided strategic input into this study and contributed genomic data. TS and CS developed AusTrakka aspects of the study. JRW, ES and KH accessed, verified and undertook data analysis, figure generation and wrote the first draft of the manuscript. All authors participated in manuscript revision and approved the final version. BH submitted the final version of the manuscript.

## Data sharing statement

The sequencing data generated in this study have been deposited to the NCBI Sequence Read Archive and corresponding accessions of Australian *Mtb* sequences used for this study are listed in Supplementary File S1.

## Editor note

The Lancet Group takes a neutral position with respect to territorial claims in published maps and institutional affiliations.

## Declaration of interests

No authors also have any financial disclosures relevant to this article. No financial or non-financial benefits have been received or will be received from any party related directly or indirectly to the individuals of this article.

## Appendix A Supplementary data

Supplementary Fig. S9

Supplememtary data, Table S1, Fig. captions
