# Supplementary material for: National genomic survey of drug-resistance and multi-jurisdictional clusters of Mycobacterium tuberculosis in Australia 2015–2023
Source: Lancet Reg Health West Pac. 2026 Jul 23;73:101928. doi: 10.1016/j.lanwpc.2026.101928 (PMC13427501; doi:10.1016/j.lanwpc.2026.101928)
Supplement: Supplememtary data, Table S1, Fig. captions [file mmc10.docx]

# Supplementary data

## Methods

**Genomic analysis**

Australian *Mtb* isolates were sequenced on Illumina platforms at PHLs, with each sequencing platform generating 150-bp paired-end reads. For core genome analysis, lineage and genomic AMR prediction (gDST), we omitted sequences where *M. tuberculosis* senso stricto was not the most abundant species or where there was evidence of contamination with non-*Mtb.* We also excluded sequences with less than 95% of Mtb genome coverage or where the mean depth was < 40X. The quality control and core genome analysis, including SNP distance calculations and generation of phylogenetic trees were conducted using the bohra microbial genomics pipeline (<https://github.com/MDU-PHL/bohra>). Specifically, identification of core genome single nucleotide polymorphisms (SNPs) against reference genome (*Mtb* H37Rv genome, GenBank accession NC 000962.3) were performed using snippy v4.4.5 (<https://github.com/tseemann/snippy>) masking repetitive sites. Core-genome alignments were used for phylogenetic reconstruction using maximum likelihood (ML) with IQtree v2.1 (constant sites corrected, 1000 bootstraps, and a generalised time-reversable model of evolution (GTR+F+G4)^13^). To identify genomic clusters, consistent with previous work, a SNP distance matrix from each core-genome alignment was created using snp-dists 0.8.2 and single-linkage clustering with thresholds of 5 SNPs was used to identify genomic clusters (<https://scikit-learn.org/stable/modules/clustering.html>)^6^.

**Detection of genomic DST**

Lineage and gDST detection were undertaken using tbtamr v1.0.3^19,20^, which implements the second edition of the WHO catalogue of mutations in *Mtb* and their association with drug resistance^21^ (variants with uncertain significance were not reported). Reporting of genomic DST profiles was performed based on the WHO guidelines for AMR in *Mtb* (Supplementary Table 1).

**Supplementary Table 1: Identification and classification of drug resistance profiles in *Mtb***

Classification of drug resistance profiles in Mtb is defined by WHO guidelines^33^.

** for the purpose of genomic classification of drug resistance profiles in a public health setting, preXDR and XDR are reported as preXDR/XDR as there is still limited data available for the mechanisms which influence resistance to delaminid, bedaquiline and linezolid in Mtb.*

| Drug resistance type | Drug resistance profile | First-line resistance mechanisms detected | Other mechanisms detected |
| --- | --- | --- | --- |
| Susceptible | First-line susceptible | No resistance mechanisms to rifampicin, isoniazid, pyrazinamide or ethambutol detected. | Not applicable |
| Mono-resistance | Isoniazid mono-resistance (HR-TB) | Resistance mechanisms to isoniazid and NOT rifampicin, ethambutol or pyrazinamide | Not applicable |
|  | Rifampicin mono-resistance (RR-TB) | Resistance mechanisms to rifampicin and NOT isonaizid, ethambutol or pyrazinamide. | No resistance to flouroquinolones detected. |
|  | Ethambutol mono-resistance | Resistance mechanisms to ethambutol and NOT rifampicin, isoniazid or pyrazinamide | Not applicable |
|  | Pyrazinamide mono-resistance | Resistance mechanisms to pyrazinamide and NOT rifampicin, isoniazid or ethambutol | Not applicable |
| Poly-resistance | Poly-resistant (RR-TB) | Resistance to rifampicin and ethambutol and/or pyrazinamide but NOT isoniazid | No resistance to flouroquinolones detected. |
|  | Poly-resistant (HR-TB) | Resistance to isoniazid and ethambutol and/or pyrazinamide but NOT rifampicin | Not applicable |
|  | Poly-resistant | Resistance to ethambutol and pyrazinamide but NOT rifampicin or isoniazid | Not applicable |
| Multi-drug resistance | MDR | Resistance to rifampicin and isoniazid | No resistance to flouroquinolones detected. |
| preXDR/XDR* | preXDR | Resistance to rifampicin and/or isoniazid | Resistance to flouroquinolones |
|  | XDR | Resistance to rifampicin and/or isoniazid | Resistance to flouroquinolones and other group A drug (bedaquiline, linezolid, delaminid) |

## Supplementary figures

**Supplementary Figure 1: Number of notifications sequenced by year and jurisdiction.** Number of notified cases (blue bars) and the number of sequences submitted (green bars) for each jurisdiction for each year of the study.

**Supplementary Figure 2: Phylogenetic lineages by jurisdiction.** Phylogenetic lineage was identified for each jurisdiction and the number of cases across the study period plotted. The stacked bars are coloured by lineage.

**Supplementary figure 3: Core genome SNP Phylogenetic trees of the *Mtb* sequences from Australia.** Core genome SNP Phylogenetic trees were generated for each lineage 1, 2, 3 or 4. Sequences which cluster together are highlighted with red nodes, and grey nodes indicate unclustered sequences.

**Supplementary Figure 4: Timeline of genomic clusters from across Australia via common Lineages 1, 2, 3 or 4.** Clusters with >= 5 cases were identified using hierarchical clustering and a 5 SNP threshold. These clusters were plotted based on the collection dates provided and coloured by the jurisdiction. The y-axis represents the cluster ID assigned for the purposes of this study.

**Supplementary Figure 5: Australian genomic clusters identified as nationally significant.** Clusters of national significance were defined and grouped into 4 categories **1)** **Large clusters (>10)** from a single jurisdiction, **2)** **Multi-jurisdictional clusters** ≥ 5 cases from > 1 jurisdiction, **3)** **Drug resistant clusters** - cluster that contains sequences with MDR from a single jurisdiction and **4) Drug resistant clusters** contain MDR from > 1 jurisdiction. Clusters which fulfil these criteria are represented on the Y axis, with the cluster ID assigned for the basis of this study, with the number of cases in those clusters from each jurisdiction displayed in bars.

**Supplementary Figure 6: Distribution of lineages in single-jurisdictional and multi-jurisdictional clusters. A)** Lineage representation of the number of clusters from lineage 1, 2 ,3 and 4 and **B)** lineage representation of the number of clustered sequences from lineage 1, 2, 3 and 4 in single and multi-jurisdictional clusters.

**Supplementary Figure 7: Median SNP distance of the *Mtb* genomic clusters.** The media SNP distance of the genomically identified clusters during the study period were determined and plotted against **A)** the duration of the cluster and **B)** the size of the clusters. Circles on the plots are coloured by jurisdiction if present in one jurisdiction or as multi-jurisdictional (MJ) if present in more than one jurisdiction.

**Supplementary figure 8: *Mtb* genomic cluster nomenclature assignment.** Addition of new sequences to a comparative analysis can cause formation of new clusters by addition of sequences to an already clustered group (cluster ID remains the same), formation of a completely new cluster containing only new sequences (new cluster ID assigned), splitting and/or merging of an existing cluster and adding new sequences (archive of existing cluster ID and assignment of new cluster ID).
